# Alpha-Glucosidase Inhibitor Voglibose Suppresses Azoxymethane-Induced Colonic Preneoplastic Lesions in Diabetic and Obese Mice

**DOI:** 10.3390/ijms21062226

**Published:** 2020-03-23

**Authors:** Junichi Kato, Yohei Shirakami, Taku Mizutani, Masaya Kubota, Hiroyasu Sakai, Takashi Ibuka, Masahito Shimizu

**Affiliations:** 1 Departments of Gastroenterology, Gifu University Graduate School of Medicine, 1-1 Yanagido, Gifu 501-1194, Japan; YFA52710@nifty.com (J.K.); taku.mizutani0912@gmail.com (T.M.); kubota-gif@umin.ac.jp (M.K.); sakaih1978@gmail.com (H.S.); tibuka-gif@umin.ac.jp (T.I.); shimim-gif@umin.ac.jp (M.S.); 2 Informative Clinical Medicine, Gifu University Graduate School of Medicine, 1-1 Yanagido, Gifu 501-1194, Japan

**Keywords:** diabetes mellitus, obesity, alpha-glucosidase inhibitor, voglibose, colorectal cancer

## Abstract

Type 2 diabetes mellitus and its related insulin resistance are known to increase the risk of cancer. Anti-diabetic agents can improve insulin resistance and may lead to the suppression of carcinogenesis. This study aimed to investigate the preventive effects of the alpha-glucosidase inhibitor voglibose on the development of azoxymethane-induced colorectal pre-neoplastic lesions in obese and diabetic C57BL/KsJ-*db/db* mice. The direct effects of voglibose on the proliferation of colorectal cancer cells were also evaluated. Mice were injected with azoxymethane to induce colorectal pre-malignancy and were then administered drinking water with or without voglibose. At the end of the study, the administration of voglibose significantly suppressed the development of colorectal neoplastic lesions. In voglibose-treated mice, serum glucose levels, oxidative stress, as well as mRNA expression of the insulin-like growth factor-1 in the colon mucosa, were reduced. The proliferation of human colorectal cancer cells was not altered by voglibose. These results suggested that voglibose suppressed colorectal carcinogenesis in a diabetes- and obesity-related colorectal cancer model, presumably by improving inflammation via the reduction of oxidative stress and suppressing of the insulin-like growth factor/insulin-like growth factor-1 receptor axis in the colonic mucosa.

## 1. Introduction

Colorectal cancer (CRC) is a serious health concern worldwide, although technologies for early detection and early treatment are progressing. Globally, CRC is the third most commonly diagnosed cancer and the fourth leading cause of cancer-related deaths in males, and the second most commonly diagnosed cancer and third leading cause of cancer-related deaths in females [1]. Therefore, to control CRC, prevention, early detection, and treatment are important.

The risk of CRC has been estimated to be 27% higher in patients with type 2 diabetes mellitus (T2DM) than in non-diabetic controls [2]. Diabetes mellitus (DM) results from malfunctions in insulin secretion and/or insulin function. T2DM is characterized by insulin resistance and relative insulin deficiency. Therefore, examination of the mechanism of diabetes and its related pathology and a more effective control of its pathological condition are expected to lead to the prevention CRC onset in patients with T2DM.

Insulin resistance accompanying hyperinsulinemia, hyperglycemia, and chronic inflammation are known mechanisms that increase cancer morbidity, especially morbidity related to colon cancer, liver cancer, and pancreatic cancer [3]. In addition to cardiovascular and cerebrovascular events, cancer is a major cause of T2DM- and obesity-related deaths [4,5,6,7].

However, it is unclear whether DM itself is the main cause of cancer or whether DM develops because obesity and dietary habit are confounding factors. Under such circumstances, in eight large cohort studies targeting more than 330,000 individuals in recent years, diabetes statistically increased the risk of cancers at specific sites, such as the colon, liver, pancreas, and bile duct. DM itself was associated with a 20% increase in the risk of cancer incidence [8]. Thus, diabetes-related conditions such as obesity and diabetes itself are risks for various cancers.

In association with insulin resistance, increases in expression levels of insulin and insulin-like growth factors (IGFs) and enhancement of signal transduction via the insulin receptor and IGF-1 receptor induce tumor cell proliferation and migration [9]. Improvements in insulin resistance may improve hyperinsulinemia and eventually suppress carcinogenesis. There are reports of drugs capable of reducing insulin resistance, thereby suppressing carcinogenesis. For example, metformin markedly reduced the incidence of gastric cancer, CRC, liver cancer, and pancreatic cancer [10].

Both insulin resistance-improving drugs and alpha-glucosidase inhibitors (AGIs) can alleviate the risk of colorectal carcinogenesis [11,12,13,14,15,16,17,18]. Several mechanisms, such as changes in bile acid patterns in feces due to the suppression of glucose absorption and reduction in neutral sterols [19], are considered, however, the clear mechanism has not yet been elucidated.

In the present study, to examine the effects of an AGI on CRC, we investigated whether the development of azoxymethane (AOM)-induced colorectal pre-neoplastic lesions in diabetic and obese *db/db* mice was suppressed by the AGI voglibose. In addition, the direct effects of voglibose on CRC cell proliferation were examined.

## 2. Results

### 2.1. General Observations

Body weight, relative liver weight, and relative white adipose tissue weight of mice treated with AOM plus voglibose were significantly decreased compared with those of mice treated with AOM alone (Table 1). During the experiment, there were no clinical symptoms in all the groups.

### 2.2. Serum Parameters

Serum alanine aminotransferase (ALT), glucose, insulin, total cholesterol, free fatty acid (FFA), and triglyceride (TG) levels in each group are listed in Table 2. The serum glucose, total cholesterol, and TG levels were significantly decreased in mice treated with AOM plus voglibose compared with those in mice treated with AOM alone. There were no other statistically significant items between Groups 2 and 4. In this study, there was no adverse effect on major serum parameters after administering voglibose.

### 2.3. AOM-Induced Colorectal Pre-Neoplastic Lesions in Experimental Mice

**Figure 1:**
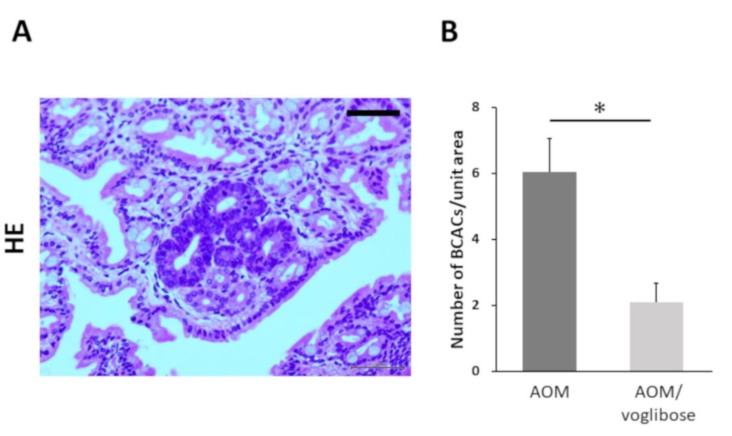
Development of BCAC in the colorectum of experimental mice. (**A**) Representative photographs of BCACs (HE staining) on the colonic mucosa of experimental mice treated with AOM. The atypical cryptal cells in BCAC have hyperchromatic nuclei and basophilic cytoplasm. The localization of accumulated β-catenin protein is apparent in the cytoplasm and nucleus of colonic atypical crypts. Scale bars, 100 µm. (**B**) Number of BCACs formed in the colorectum. The values are expressed as mean ± SE. Asterisk, statistical significance (*p* < 0.05).

Colorectal pre-neoplastic lesions, β-catenin-accumulated crypts (BCACs) [20,21], developed in the colon of AOM-injected mice (mice treated with AOM alone and mice treated with AOM plus voglibose). Optical microscopic representative photographs of AOM-induced BCACs are shown in Figure 1A. The total number of BCACs were significantly less in mice treated with AOM plus voglibose compared with that in mice treated with AOM alone (Figure 1B). The incidence of BCACs in mice treated with AOM alone was 91.6% and that in mice treated with AOM plus voglibose was 58.3%. These results suggest that voglibose has an inhibitory effect on the development of colorectal pre-neoplastic lesions.

### 2.4. Effects of Voglibose on Antioxidant Activity and Inflammatory Cytokines

**Figure 2:**
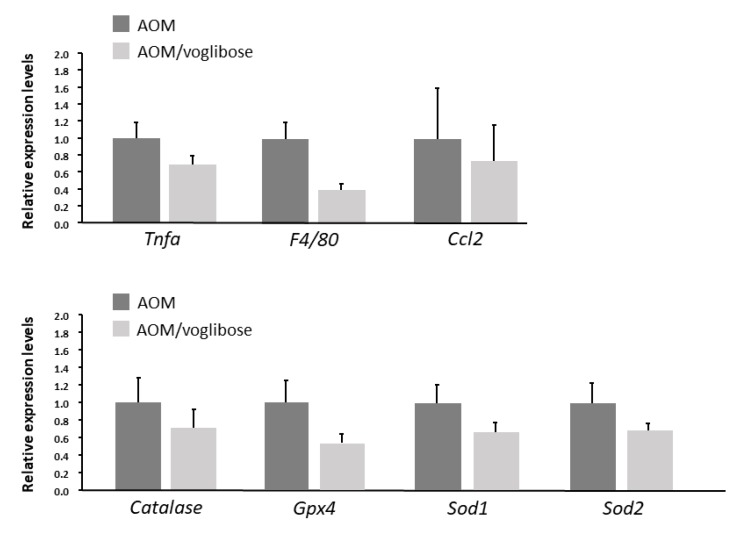
mRNA expression levels of inflammatory cytokines and anti-oxidative enzymes in colonic mucosa of experimental mice. Relative expression levels of mRNA of Tnf-a, F4/80, Ccl-2, catalase, Gpx-4, Sod-1, and Sod-2 measured by qRT-PCR. 18S was used as the internal control. Data represent means ± S.E. from the various experimental groups. AOM, azoxymethane.

**Figure 3:**
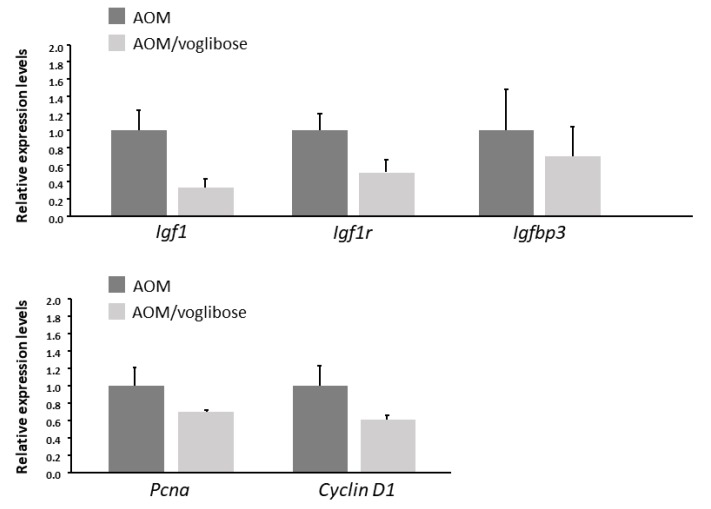
mRNA expression levels of proteins related to cell proliferation in the colonic mucosa of experimental mice. Relative expression levels of mRNA of IGF-1, IGF-1R, IGFBP-3, pcna, and cyclin d1 measured by qRT-PCR. 18S was used as the internal control. Data represent means ± S.E. from the various experimental groups. AOM, azoxymethane.

The effects of voglibose on mRNA expression levels of specific molecules, such as cyclooxygenase-2 (Cox-2), tumor necrosis factor-α (Tnf-a), F4/80, C-C motif chemokine-2 (Ccl-2), catalase, glutathione peroxidase-4 (Gpx-4), superoxide dismutase-1 (Sod-1), Sod-2, Igf-1, Igf-2, IGF-binding protein-3 (Igfbp-3), *Pcna*, and *Cyclin d1*, associated with antioxidant activity, colonic inflammation, and cell-cycle, were examined by quantitative real-time reverse transcription-polymerase chain reaction (qRT-PCR) analysis. mRNA expression of these genes was not statistically significantly different between the experimental groups (Figure 2 and Figure 3). However, there was a decreased trend in mRNA expression levels of F4/80 and IGF-1 in mice treated with AOM plus voglibose compared with those in mice treated with AOM alone.

### 2.5. Effects of Voglibose on NF-κB Activity in the Colonic Mucosa of Experimental Mice

**Figure 4:**
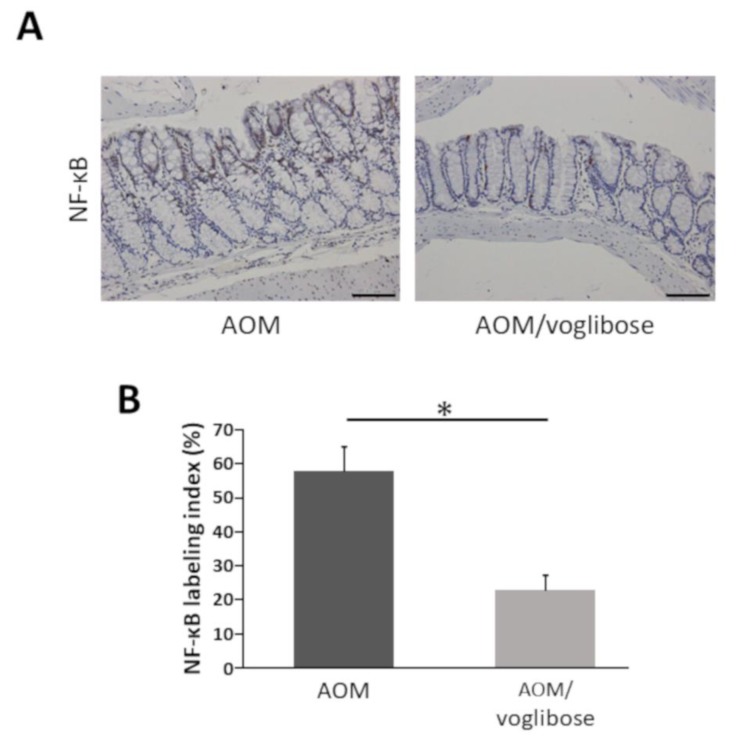
Effects of voglibose on the NF-κB activity in the colonic mucosa of experimental mice. Sections of the colon were stained with anti-phospho-NF-κB p65. (**A**) Representative photographs from each group, mice treated with AOM alone and mice treated with AOM plus voglibose, are shown. (**B**) The positive cell indices, which were determined by counting phospho-NF-κB p65-positive cells, are shown. Bars, 100 μm. Asterisk, statistical significance (*p* < 0.05).

NF-κB is critically associated with progression of inflammation and cell proliferation in colonic mucosa [22]. Therefore, the effects of voglibose on NF-κB activity in the colonic mucosa was investigated. The indices of phospho-NF-κB p65-positive cells were markedly reduced by treatment of voglibose (Figure 4). The finding indicates that voglibose significantly inhibits NF-κB activity and attenuates the inflammation in the colonic mucosa of AOM-treated *db/db* mice.

### 2.6. Effects of Voglibose on Systemic Oxidative Stress Levels in Experimental Mice

**Figure 5:**
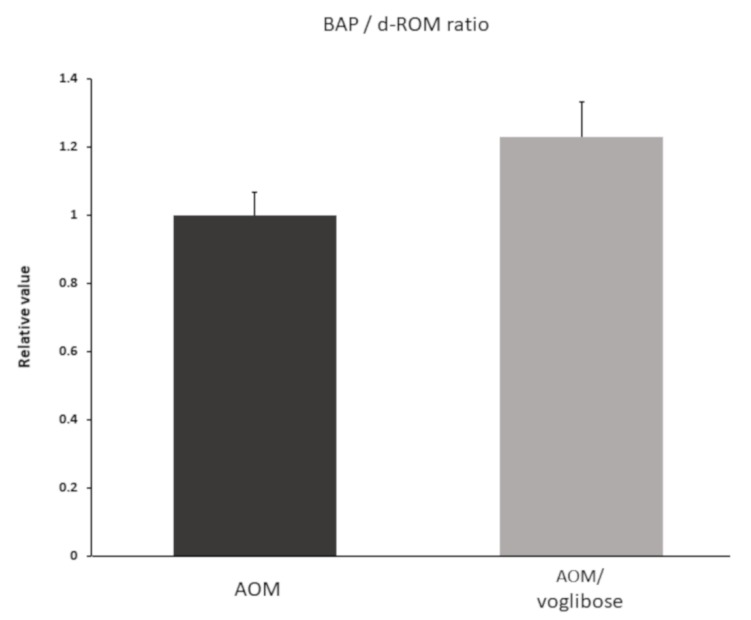
Serum reactive oxygen metabolites and biological antioxidant potential of experimental mice. d-ROM and BAP were measured one week after the mice were killed. Data shown as BAP/d-ROM ratio. Data represent means ± S.E. AOM, azoxymethane.

To investigate systemic oxidative stress, reactive oxygen metabolites (d-ROMs) test and biological antioxidant potential (BAP) test were performed. Data are shown as BAP/d-ROM ratio (Figure 5). Although there was no significant difference, BAP/d-ROM ratio was higher in mice treated with AOM plus voglibose than in mice treated with AOM alone.

### 2.7. Effects of Voglibose on Cellular Proliferation in CRC Cells

**Figure 6:**
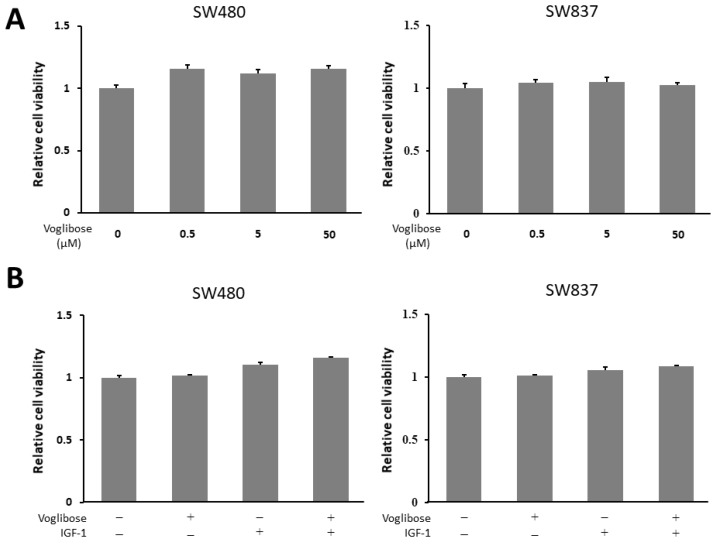
Effects of voglibose on cellular proliferation in SW480 and SW837 cells. (**A**) Cell proliferation assay of SW480 and SW837 cells treated with different levels of voglibose after 72-h in vitro incubation. (**B**) Cell proliferation assay of SW480 and SW837 cells treated with voglibose (50 µM) and/or IGF-1 (10 nM) after 72-h incubation. Data represent means ± S.E. from the various experimental groups. IGF-1, insulin-like growth factor -1.

To evaluate whether voglibose directly inhibits the growth of CRC cells, we performed a cell proliferation assay in human CRC cell lines (SW480 and SW837 cells). The 3-(4,5-dimethylthiazol-2-yl)- 5-(3-carboxymethoxyphenyl)- 2-(4-sulfophenyl)- 2H-tetrazolium (MTS) assay demonstrated that both high and low levels of voglibose showed no significant alteration in the proliferation of SW480 and SW837 cells (Figure 6A). Next, to evaluate whether voglibose has an inhibitory effect on CRC cell proliferations in the presence of IGF-1, the MTS assay was performed in the same manner with 50 µM of voglibose and 10 nM of IGF-1 with a culture time of 72 h. Results showed that voglibose had no significant effect on the proliferation of IGF-1-stimulated SW480 and SW837 cells (Figure 6B).

## 3. Discussion

DM is associated with a greater risk of CRC. According to a meta-analysis, the overall hazard ratio for CRC-specific incidence was 1.26 and that for CRC-specific mortality was 1.38 in patients with DM compared to those without DM [23]. As site specific risk factors, it was reported that age, female gender, black, non-Hispanic race, and the presence of comorbidities, including diabetes, were associated with developing colon cancer in a proximal location [24]. Recent prospective study also indicated an increased risk of proximal colon cancer in women with T2DM [25]. CRC shares some cellular and molecular pathways (epithelial cell injury, activation of inflammation, Wnt/β-catenin pathways, and iron homeostasis defects) with diabetes targeting organ damage, such as kidneys [26]. Recent research has focused on investigating the carcinogenesis inhibitory properties of drugs used for treating DM. For example, metformin (biguanide) is the drug used to treat T2DM and it reduces the risk of CRC-specific death by 34% in CRC patients compared with that in non-users [2]. In addition, tofogliflozin (sodium-glucose cotransporter 2 inhibitor) is the drug used to treat T2DM and it reduces the risk of CRC through attenuation of chronic inflammation, hyperglycemic state and IGF/IGF-1R axis activation in obesity and diabetes mice [27].

Carbohydrates are normally converted into simple sugars (monosaccharides) by alpha-glucosidase enzymes present on cells lining the intestine, enabling monosaccharides, such as glucose, to be absorbed through the intestine. AGIs are oral anti-diabetic drugs used for T2DM that function by inhibiting several alpha-glucosidase enzymes, thus preventing the digestion of carbohydrates. In a recent study, it was suggested that the therapeutic effects of AGIs are not only based on a delayed digestion of complex carbohydrates but also on metabolic effects of colonic starch fermentation [28]. Several epidemiological and basic medical studies have revealed that T2DM is as an independent risk factor for CRC [18] and that AGIs may reduce the risk of CRC [29]. The risk of CRC development in diabetes patients was reduced by taking acarbose, an AGI, in a cohort study in Taiwan [30] Among the pathophysiological conditions induced by T2DM, insulin resistance and hyperinsulinemia might be related to colorectal carcinogenesis. Improvement of these events is an effective strategy for suppressing CRC development [15,16]. The use of AGIs is reported to improve hyperinsulinemia [13] by delaying carbohydrate absorption in the small intestine and preventing a rapid increase in the blood glucose level [12]. This might be a key mechanism underlying its suppression of colorectal neoplasia.

In the present study, we investigated the preventive effects of the AGI voglibose on the development of AOM-induced colorectal pre-neoplastic lesions in obese and diabetic *db/db* mice. Previous reports suggested carcinogen AOM could develop adenoma and following adenocarcinoma in mice in accordance with the adenoma-carcinoma sequence [31,32], although the duration of the present study was relatively short and these lesions were not observed. In addition, obese and diabetic mice were susceptible to AOM-induced colonic lesions [33]. Therefore, this rodent model was considered suitable for studying obesity/diabetes-related colorectal tumorigenesis and has been previously employed in many examinations [34].

**Figure 7:**
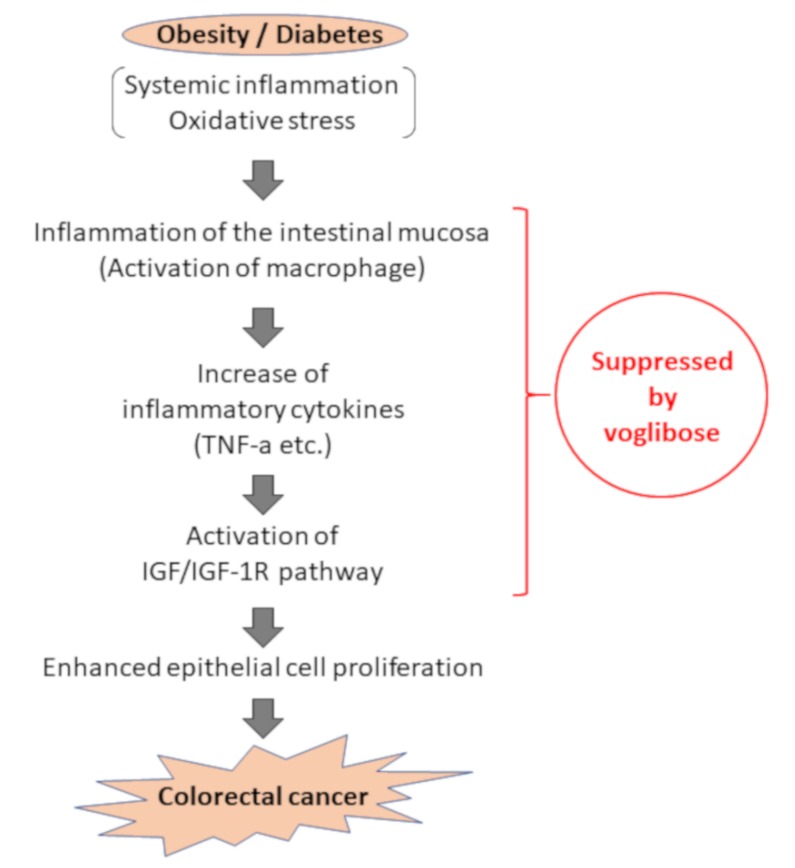
Assumed mechanism of inhibitory effect of voglibose on colorectal carcinogenesis due to obesity and diabetes pathology. In the pathology of obesity/diabetes, inflammation in the intestinal mucosa is formed by activation of inflammatory cells, including macrophages, against the background of systemic and chronic inflammation and oxidative stress, and an increase of inflammatory cytokines such as TNF-α. Accordingly, activation of the IGF/IGF-1R signaling pathway occurs, and cell proliferation of intestinal mucosal epithelial cells is enhanced, leading to carcinogenesis. This study suggests that voglibose can suppress colorectal carcinogenesis through suppression of intestinal inflammation and IGF/IGF-1R signaling pathway.

We evaluated the expression levels of various mRNAs of colonic mucosal epithelial cells and found that there was a decreased trend in the mRNA expression levels of F4/80 and IGF-1 in mice treated with AOM plus voglibose compared with that in mice treated with AOM alone. Decreased levels of F4/80 suggests that voglibose improves inflammation in the colonic mucosa. Reduced NF-κB indices in the colonic mucosa also indicated that voglibose appeared to attenuate mucosal inflammation in AOM-treated *db/db* mice. When chronic colonic mucosal inflammation is preceded, Wnt signaling is activated in the regenerating mucosa. Based on the inflammatory microenvironment, a mutation in the tumor growth factor β gene further causes colon cancer without going through adenoma [35]. Therefore, it is speculated that the reduction of colorectal mucosal inflammation leads to the reduction of colorectal carcinogenesis. Decreased levels of IGF-1 and IGF-1R suggested that voglibose inhibits the activation of IGF-1R by decreasing serum levels of IGF-1. Recent studies have suggested that the activation of the IGF/IGF-1R axis stabilizes and activates the Wnt/β-catenin pathway involved in colorectal carcinogenesis [36,37]. Especially in obesity-related colorectal carcinogenesis, a relationship between upregulation of the IGF/IGF-1R axis and the accumulation of β-catenin was observed [16]. Therefore, it is recognized that the IGF/IGF-1R system is involved in colorectal carcinogenesis and therefore may be a potential molecular target that could help in the prevention and treatment of CRC [16,38,39,40]. Hence, this study suggests that voglibose has an inhibitory effect in colorectal carcinogenesis via the suppression of the IGF/IGF-1R axis (Figure 7).

Focusing on these results, the effect of voglibose on cell proliferation by IGF-1 was examined using a human colon cancer cell line. In a previous study comparing the expression levels of IGF-1R protein in Caco2, HT29, SW480, and SW837 cell lines by western blot analysis, the expression level of the IGF-1R protein was shown to be significantly increased in SW480 and SW837 cell lines [41]. Based on this study, we decided to perform a cell proliferation assay using SW480 and SW837 cell lines. The MTS assay showed that voglibose did not inhibit the proliferation of SW480 or SW837 cells with or without IGF-1. These results suggest that voglibose does not directly inhibit the proliferation of CRC cells.

In this study, the BAP/d-ROM ratio was higher in mice treated with AOM plus voglibose than in mice treated with AOM alone. Recent studies have shown that a glucose spike activates inflammatory markers and promotes superoxide production [42]. It has been reported that the generation of reactive oxygen species associated with postprandial hyperglycemia is a potential mechanism that impairs endothelial function [43]. Abnormal accumulation of highly reactive oxides such as reactive oxygen species in the intestinal mucosa can cause intestinal inflammation such as inflammatory bowel disease. Therefore, the prevention of postprandial hyperglycemia by AGIs such as voglibose is considered to have a potential anti-inflammatory effect by reducing oxidative stress in the intestinal mucosa.

One of the side effects of AGIs is intestinal dysfunction. Although constipation is the most common side effect, diarrhea tends to be a relatively common example. Focusing on diarrhea as a side effect, a clinical study showed that AGI therapy reduces the bowel transit time of stool in diabetes patients—especially in frail elderly and immobile patients—experiencing constipation [14]. This side effect of AGIs may play a role in colorectal tumorigenesis because the long-term exposure to bile acids due to delayed stool transit plays a role in CRC development [11]. In addition, AGIs are reported to increase the levels of butyrate, which is associated with the inhibited growth of transformed cells in the colorectal mucosa [17] and the inhibition of the production and activity of bile acids, particularly secondary bile acids, which are said to have carcinogenic effects owing to weak acidification of the intestinal environment. Moreover, many studies have indicated the links between gut microbiota and CRC development [44], and recent investigations have shown that AGI may attenuate inflammation and ameliorate DM through modulating intestinal microbiota [45,46]. Those effects of AGIs might be associated with suppression of CRC development, although bile acid metabolism or alteration of gut microbiota were not examined in this study.

## 4. Materials and Methods

### 4.1. Animals and Housing Conditions

Thirty-six male *db/db* mice (5 weeks old) were obtained from Japan SLC Inc. (Shizuoka, Japan). They were acclimatized for a week before initiating the experiment [16]. They were carefully handled and humanely maintained at the Gifu University Life Science Research Center in accordance with the Institutional Animal Care Guidelines (the authorization code 30-7 on 5 April 2018). The mice were housed in cages and kept on a basic diet of CE-2 obtained from Oriental Yeast (Tokyo, Japan) and sterilized tap water ad libitum. The environmental conditions inside the room were as follows: temperature, 23 ± 1 °C; humidity, 50% ± 20%; and 12-h light/dark cycle.

### 4.2. Chemicals and Methods of Drug Administration

AOM was obtained from Wako Pure Chemical Co. (Osaka, Japan) and was injected intraperitoneally once weekly from 1 to 4 weeks after initiating the experiment. AOM was diluted with ultra-pure water to administer a dose of 15 mg/kg body weight. Voglibose (10 mg/kg body weight; Waco Pure Chemical Co., Osaka, Japan) was dissolved in ultra-pure water. The solution was poured into bottles and was replaced with fresh solution twice a week.

### 4.3. Procedure for Animal Experiment

At five weeks of age, the mice were randomly divided into four experimental groups and treated as follows: no treatment (Group 1, *n* = 6), AOM alone (Group 2, *n* = 12), voglibose alone (Group 3, *n* = 6), and AOM followed by voglibose (10 mg/kg body weight) in the drinking water (Group 4, *n* = 12). Group 2 and 4 mice were injected with AOM (15 mg/kg body weight) as described above. The mice were not anesthetized before AOM injection. At 9 weeks of age, Group 3 and 4 mice received drinking water containing voglibose. At 24 weeks of age, counting from 14 weeks of voglibose treatment, all the mice were killed after 15 h of fasting. Blood samples were collected from the inferior vena cava for clinical chemical examinations, and organs and tissues were removed for histopathological examinations. To kill the mice, CO2 inhalation was used, after which the chest cavity was opened, and the heart was exposed and viewed directly to confirm death. The experimental protocol was approved by the Committee of Institutional Animal Experiments of Gifu University (authorization code: 30-7, dated April 13, 2018).

### 4.4. Histopathological Examination

The livers, kidneys, white adipose tissues, and colorectal tissues were excised. The colon and rectum were opened longitudinally and fixed on a filter paper in 10% buffered formalin for more than 24 h. It was then divided into a rectal portion (approximately 1 cm in length on the oral side from the dentate line) and a colonic portion. Each segment and the other tissues were paraffin embedded. From the rectal tissue blocks, two serial sections were subjected to hematoxylin and eosin (HE) staining for histopathology to evaluate the development of BCACs [16]. Immunohistochemical staining for phospho-nuclear factor-κB (NF-κB) p65 was run on histological sections to estimate NF-κB activity, respectively, in the colonic crypts [11,26], using the LSAB Kit (DAKO, Glostrup, Denmark) with primary antibody, anti-phospho-NFκB p65 antibody (a final dilution of 1:50, Ser276; Cell Signaling Technology, Danvers, MA, USA). The positive cell index (%) for phospho-NF-κB p65 was determined based on previous methods [15,47].

### 4.5. Clinical Chemical Examination

Blood samples were collected from the inferior vena cava of the mice. Whole blood serum was centrifuged and used for chemical analyses. Serum ALT, glucose, total cholesterol, FFA, and TG levels were determined by a commercial laboratory (SRL, Inc., Japan). To investigate systemic oxidative stress, oxidative activity was assessed by measuring d-ROMs [48] and anti-oxidative activity was assessed by measuring BAP [49] using FREE Carpe Diem (Diacron International s.r.l., Grosseto, Italy) in accordance with the manufacturer’s protocol.

### 4.6. mRNA Extraction and qRT-PCR Analysis

mRNA expression in the colonic mucosa of the experimental mice was examined using qRT-PCR analysis. Total RNA was isolated from the scraped colonic mucosa of all experimental mice using PureLinkTM RNA Mini Kit (Invitrogen, Carlsbad, CA, USA) according to the manufacturer’s instructions. Complementary DNA (cDNA) was amplified from 1.5 μg of total RNA from each sample using High-Capacity cDNA Reverse Transcription Kit (Applied Biosystems, Foster City, CA, USA). The primers used for amplifying Cox-2, Ccl-2, Tnf-a, F4/80, catalase, Gpx-4, Sod-1, Sod-2, Igf-1, Igf-2, Igfbp-3, and 18S-specific genes were previously reported [50,51] or as follows: *Igf-1*, forward 5′-TCG GCC TCA TAG TAC CCA CT-3′, reverse 5′-ACG ACA TGA TGT GTA TCT TTA TTG C-3′; *Igf-2*, forward 5′-ACC TTC GGC CTT TGT CTG GTA-3′, reverse 5′-CGA AGG CCA AAG AGA TGA GA-3′; and *Igfbp-3* forward 5′-GAC GAC GTA CAT TGC CTC AG-3′, reverse 5′-GTC TTT TGT GCA AAA TAA GGC ATA-3′. Real-time PCR was performed using a Light Cycler (Roche Diagnostics Co., Indianapolis, IN, USA) with FastStart Essential DNA Green Master (Roche Diagnostics Co., Indianapolis, IN, USA). The expression of these genes was normalized to *18S*.

### 4.7. Cell Lines and Culture Conditions

SW837 and SW480 human CRC cell lines and a Huh7 human hepatoma cell line were used. Huh7 was used as a positive control [52]. These cell lines were obtained from the JCRB Cell Bank (Osaka, Japan). All cell lines were authenticated via short tandem repeat analysis by PCR and were certified to be free of bacteria, fungi and mycoplasmas by the cell bank. These cell lines were grown in appropriate media according to the instructions of cell bank. Cells were subjected to experiments within 6 months after receipt. All cells were maintained in Dulbecco’s modified Eagle’s medium (Sigma-Aldrich, Saint Louis, MO, USA) supplemented with 10% fetal bovine serum (Sigma-Aldrich, St. Louis, MO, USA) and 1% penicillin/streptomycin (Sigma-Aldrich, St. Louis, MO, USA) in an incubator at 37 °C under a humidified atmosphere containing 5% CO2.

### 4.8. Cell Proliferation Assays

SW480 and SW837 cells were seeded in 96-well plates. On the following day, cells were treated with varying levels (0, 0.5, 5, and 50 µM) of voglibose for 72 h. Cell proliferation assays were performed using MTS assay (Promega, Madison, WI, USA) in accordance with the manufacturer’s protocol.

To evaluate the effect of voglibose on cell proliferation in the presence of IGF-1, an MTS assay was performed in the same manner. SW480 and SW837 cells were seeded at a density of 3 × 10^3^ cells per well in 96-well plates in RPMI-1640 medium supplemented with 10% fetal bovine serum and 1% penicillin/streptomycin. After a 24-h incubation, the cells were treated with the medium containing ultra-pure water (solvent control), IGF-1 (10 nM), and/or voglibose (50 µM). After 72-h incubation, the effects of the drug on cell proliferation were evaluated using the MTS assay in accordance with the manufacturer’s protocol.

### 4.9. Statistical Analyses

Data are represented as mean ± standard error (SE). Differences between groups were analyzed using the Kruskal-Wallis test and then the Steel-Dwass test was performed between the groups on items to confirm statistical significance. A *p*-value of <0.05 was considered statistically significant.

## 5. Conclusions

The risk of CRC is increased by diabetes, obesity, and their related metabolic abnormalities such as chronic inflammation and a hyperglycemic state. These abnormalities might be effective therapeutic targets for preventing CRC. This study demonstrated that the administration of the AGI voglibose suppressed colorectal carcinogenesis in AOM-injected *db/db* mice, presumably by improving inflammation via the reduction of oxidative stress and suppressing of the IGF/IGF-1R axis in the colonic mucosa. However, a direct antitumor effect on CRC cell lines could not be confirmed. Further studies should investigate whether this class of drug can be used for CRC chemoprevention in individuals with diabetes.

## Figures and Tables

**Table 1:** General observations of the experimental mice.

Group	Treatment	Number of Mice	Body Weight (g)	Relative Weight (g/100 g Body Weight) of:		
				Liver	Kidneys	White Adipose Tissue
1	No treatment	6	41.4 ± 5.1^*^	6.9 ± 0.5^‡^	1.4 ± 0.2	5.1 ± 0.4
2	AOM	12	46.8 ± 0.7	4.9 ± 0.1	1.0 ± 0.1	5.9 ± 0.2
3	Voglibose	6	48.5 ± 1.8	4.6 ± 0.3^†^	0.9 ± 0.1	5.1 ± 0.3
4	AOM + Voglibose	12	43.1 ± 1.0^‡^	3.9 ± 0.1^†‡^	1.0 ± 0.1	4.7 ± 0.1^‡^

* Mean ± SE. **^†^** Significantly different from group 1 (*p* < 0.05). **^‡^** Significantly different from group 2 (*p* < 0.05). AOM, azoxymethane.

**Table 2:** Serum parameters of the experimental mice.

Group	Treatment	ALT	Glucose	Insulin	Total Cholesterol	FFA	TG
		(IU/L)	(mg/dL)	(ng/mL)	(mg/dL)	(µEQ/L)	(mg/dL)
1	No treatment	11.3 ± 3.1^*^	294.2 ± 31.7	4.4 ± 0.4	21.0 ± 3.3	246.2 ± 52.7	6.5 ± 0.9
2	AOM	15.7 ± 1.3	327.1 ± 30.2	2.1 ± 0.4^†^	13.3 ± 0.4	340.1 ± 17.0	5.0 ±0.7
3	Voglibose	8.2 ± 1.2^‡^	265.7 ± 34.0	3.4 ± 0.5	10.3 ± 0.7^†‡^	315.7 ± 16.3	4.5 ± 0.6
4	AOM + Voglibose	15.3 ± 2.6	170.2 ± 28.9^‡^	6.9 ± 2.6	10.9 ± 1.0^†‡^	290.3 ± 12.4	2.9 ± 0.3^‡^

* Mean ± SE. **^†^** Significantly different from group 1 (*p* < 0.05). **^‡^** Significantly different from group 2 (*p* < 0.05). AOM, azoxymethane; ALT, alanine aminotransferase; FFA, free fatty acid; TG, triglyceride.
